# Comparison of interleukin-2-inducible kinase (ITK) inhibitors and potential for combination therapies for T-cell lymphoma

**DOI:** 10.1038/s41598-018-32634-5

**Published:** 2018-09-21

**Authors:** Sami Mamand, Rebecca L. Allchin, Matthew J. Ahearne, Simon D. Wagner

**Affiliations:** Leicester Cancer Research Centre and Ernest and Helen Scott Haematological Research Institute, University of Leicester, Leicester, LE1 7HB UK

## Abstract

Patients with peripheral T-cell lymphomas generally have poor clinical outcomes with conventional chemotherapy. Recent advances have demonstrated that a large subgroup of PTCL are derived from follicular helper (Tfh) T-cells. These cases show a characteristic pattern of gene expression, which includes high-level protein expression of interleukin-2-inducible kinase (ITK). ITK is a member of the TEC family of kinases and normally has essential functions in regulating T-cell receptor signalling and T-cell differentiation. Here we report a side-by-side comparison of four ITK inhibitors. We investigate effects on apoptosis, phosphorylation of signaling molecules, calcium flux and migration. In line with a specific mechanism of action ONO7790500 and BMS509744 did not inhibit MEK1/2 or AKT phosphorylation although other ITK inhibitors, ibrutinib and PF-06465469, did have this effect. Specific ITKi had modest effects on apoptosis alone but there was definite synergy with doxorubicin, pictilisib (PI3Ki) and idelalisib (PI3Kδi). ITKi repressed migration of Jurkat cells caused by CXCL12 and the CXCR4 antagonist, plerixafor enhanced this effect. Overall ITKi may have several mechanisms of action that will be therapeutically useful in PTCL including reduction in survival and perturbation of trafficking.

## Introduction

Peripheral T-cell lymphomas (PTCL) are a diverse group of diseases accounting for about 5% of all non-Hodgkin’s lymphomas. With the exception of ALK^+^ anaplastic large cell lymphoma (ALCL) they have a poor clinical outlook with a 25 to 30% 5-year overall survival^1^. Standard first line treatment is with combination chemotherapy^2–4^ but the majority of patients relapse. There is no standard treatment for refractory or relapsed disease but histone deacetylase inhibitors, panabinostat and romidepsin, and pralatrexate are licensed for use in the United States^5–8^. There is also no consensus on the place of intensified treatments including stem cell transplant^9–11^. While autologous stem cell transplant is feasible only a minority of patients are suitable and there is no convincing evidence for clinical benefit.

There is, therefore, a need for new treatments. Over the past few years gene expression analysis^12,13^ and sequencing studies have transformed understanding of the biology of PTCL. Angioimmunoblastic T-cell lymphoma (AITL) and about 20% of PTCL-not otherwise specified (PTCL-NOS) have similar gene expression signatures to the normal CD4^+^ T-cell subset follicular helper (Tfh) T-cells. Tfh cells are characterised by surface expression of PD-1, CXCR5 and inducible co-stimulator (ICOS) and nuclear expression of BCL6. They are required for normal germinal centre responses and drive B-cell proliferation in part through production of IL-4 and IL-21^14^. This finding has clarified some of the clinical findings associated with AITL such as the association with paraprotein production, extensive B-cell infiltrate and sometimes the development of B-cell lymphomas. The mutational landscape of PTCL is also becoming clearer: a point mutation causes the replacement of glycine by valine at residue 17 of RHOA in 60 to 70% of cases of AITL and about 20% of PTCL-NOS suggesting a new biologically based category of Tfh lymphoma. Mutations in epigenetic modifiers^15–17^ and T-cell receptor signalling molecules^18^ are also being characterised.

Interleukin-2-inducible kinase (ITK) is a T-cell specific tyrosine kinase^19^, which is essential for signalling from the T-cell receptor (TCR)^20,21^ and also for chemokine induced migration^22,23^. Mice bearing homozygous disruptions of ITK show defects in CD4^+^ T-cell differentiation^24–28^.

ITK is a tyrosine kinase expressed in AITL^29^ and a chromosomal translocation involving ITK and SYK^30^ is present in about 20% of follicular T-cell lymphoma^31^ and is sufficient to drive lymphomagenesis in a mouse model^32^.

ITK has a structurally similar ATP binding site to that of the B-cell specific tyrosine kinase, BTK^33^ and ibrutinib, a small molecule BTK inhibitor that is clinically effective in various B-cell lymphoproliferative diseases, also inhibits ITK. This has suggested that ibrutinib might find uses in treating T-cell diseases^33,34^. Ibrutinib is currently being trialled in PTCL (clinicaltrials.gov, NCT02309580) but it is likely to reach optimal efficacy in combination with other agents, either conventional chemotherapy or novel small molecule inhibitors. Here we determine synergistic combinations with ibrutinib or ONO-7790500 a highly specific ITK inhibitor, and report potential enhancement of activity to be taken forward in further pre-clinical testing in mouse models of T-cell lymphoma.

## Results

### Characterisation of ONO-7790500: effects on growth, motility and cytokine production

**Figure 1:**
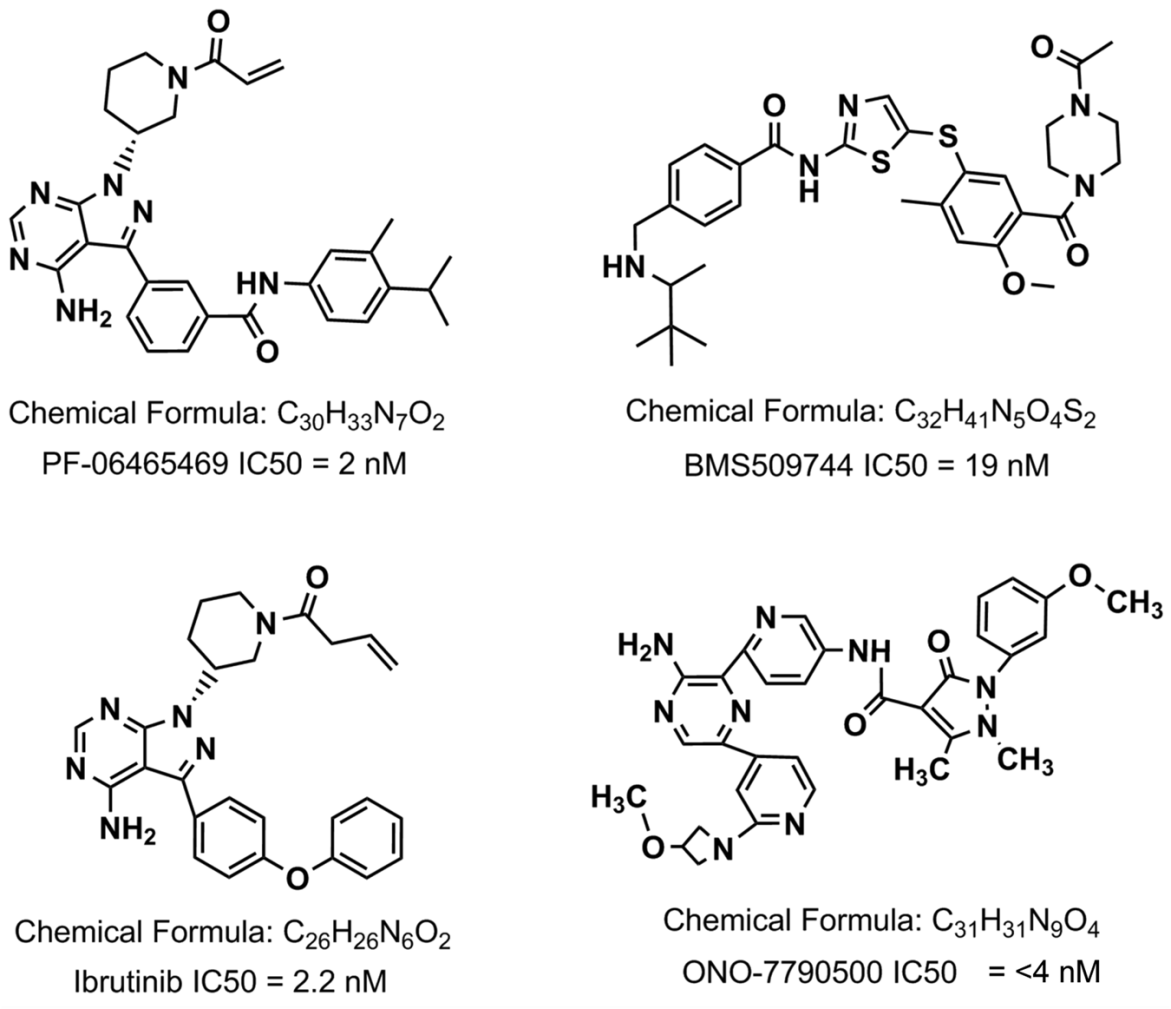
Chemical structures of ITK inhibitors used in the studies. The chemical formula and IC50 for the purified enzyme are presented beneath the structures.

Four human T-cell lines were selected for study: Jurkat, MOLT4, CCRF CEM are derived from acute lymphoblastic leukaemia and K299 from a patient with anaplastic large cell lymphoma. Jurkat has been extensively used for the analysis of T-cell receptor and calcium signalling as well as cytokine production^35^. We compared four small molecule ITK inhibitors: ONO-7790500 (IC50 <4 nM), BMS-509744 (IC50 19 nM), PF-06465469 (IC50 2 nM) and ibrutinib (IC50 2.2 nM) (Fig. 1).

**Figure 2:**
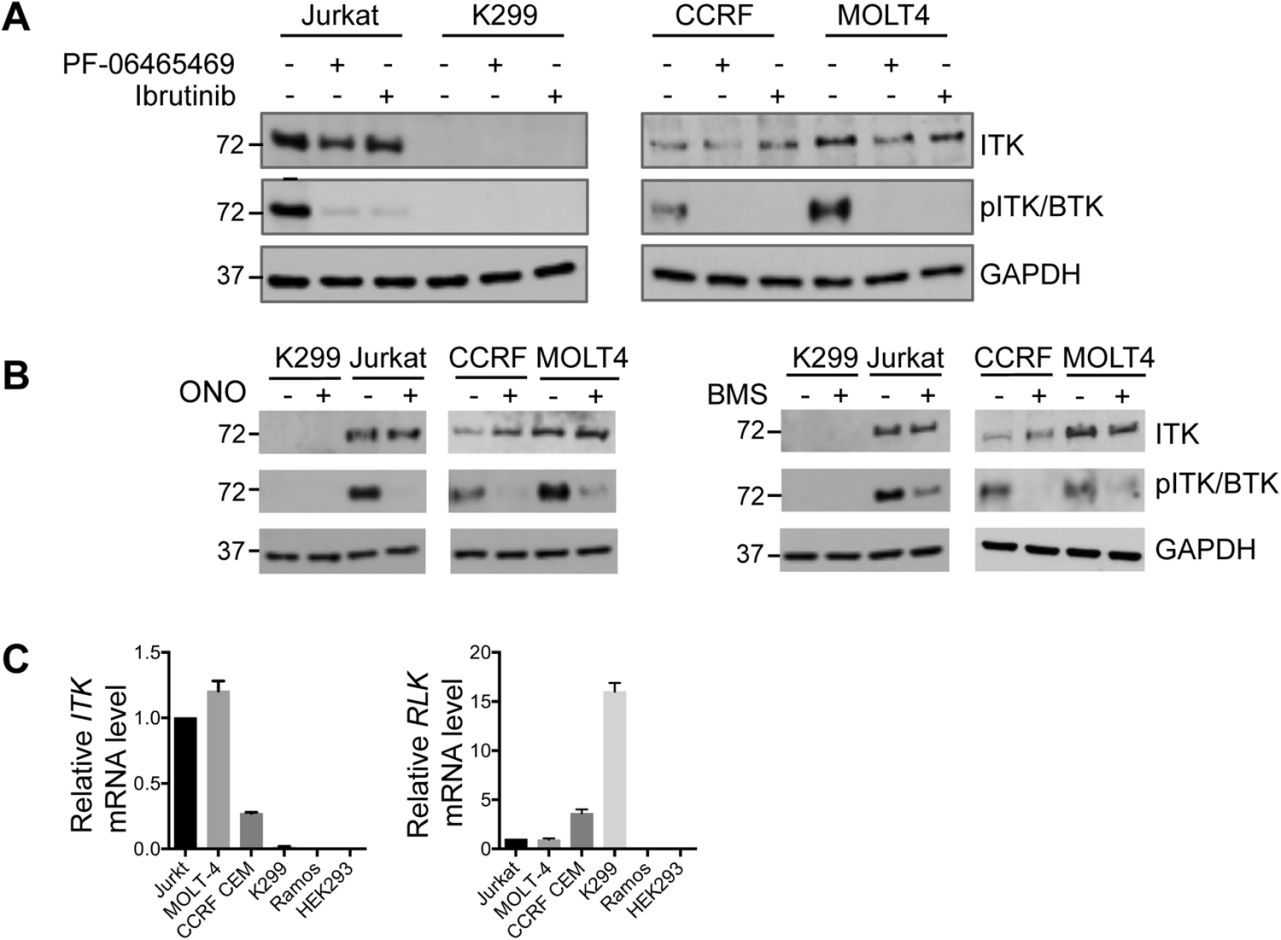
ITK expression in four T-cell lines. (**A**) Westerns of lysates from Jurkat, MOLT4, CCRF CEM and K299 showing expression of ITK and phosphorylated ITK (p-ITK) at baseline and following treatment with ITK inhibitors PF-06465469 (2 µM) and ibrutinib (2 µM) for 24 hours. GAPDH is a loading control. (**B**) Westerns of lysates from the four cell lines showing expression of ITK and phosphorylated ITK (p-ITK) at baseline and following treatment with ITK inhibitors ONO7790500 (2 µM) and BMS509744 (2 µM) for 24 hours. GAPDH is a loading control. Molecular weight is indicated to the left of the western. (**C**) Relative mRNA levels of ITK and RLK in Jurkat, MOLT4, CCRF CEM and K299. Levels are relative to Jurkat cells.

ITK protein was expressed in Jurkat, MOLT4 and CCRF CEM but was not detectable in K299 (Fig. 2A,B). All four of the small molecule ITK inhibitors (ITKi) reduced ITK phosphorylation although BMS509744 appeared less potent than the other inhibitors in Jurkat cells. To further characterise ITK in these cell lines we determined relative mRNA expression. Jurkat and MOLT4 had similar levels of *ITK* while CCRF CEM had levels about 25% of those in Jurkat and in K299 expression was undetectable (Fig. 2C). Of the TEC family kinases T-cell express very little TEC (~100-fold less than ITK^36^) but RLK (~3-fold less than ITK^36^), has been suggested to substitute for some ITK functions. Accordingly we assessed *RLK* mRNA levels. Compared to Jurkat and MOLT4, which had similar *RLK* levels, K299 had levels ~15-fold higher and CCRF CEM levels ~4-fold higher.

**Figure 3:**
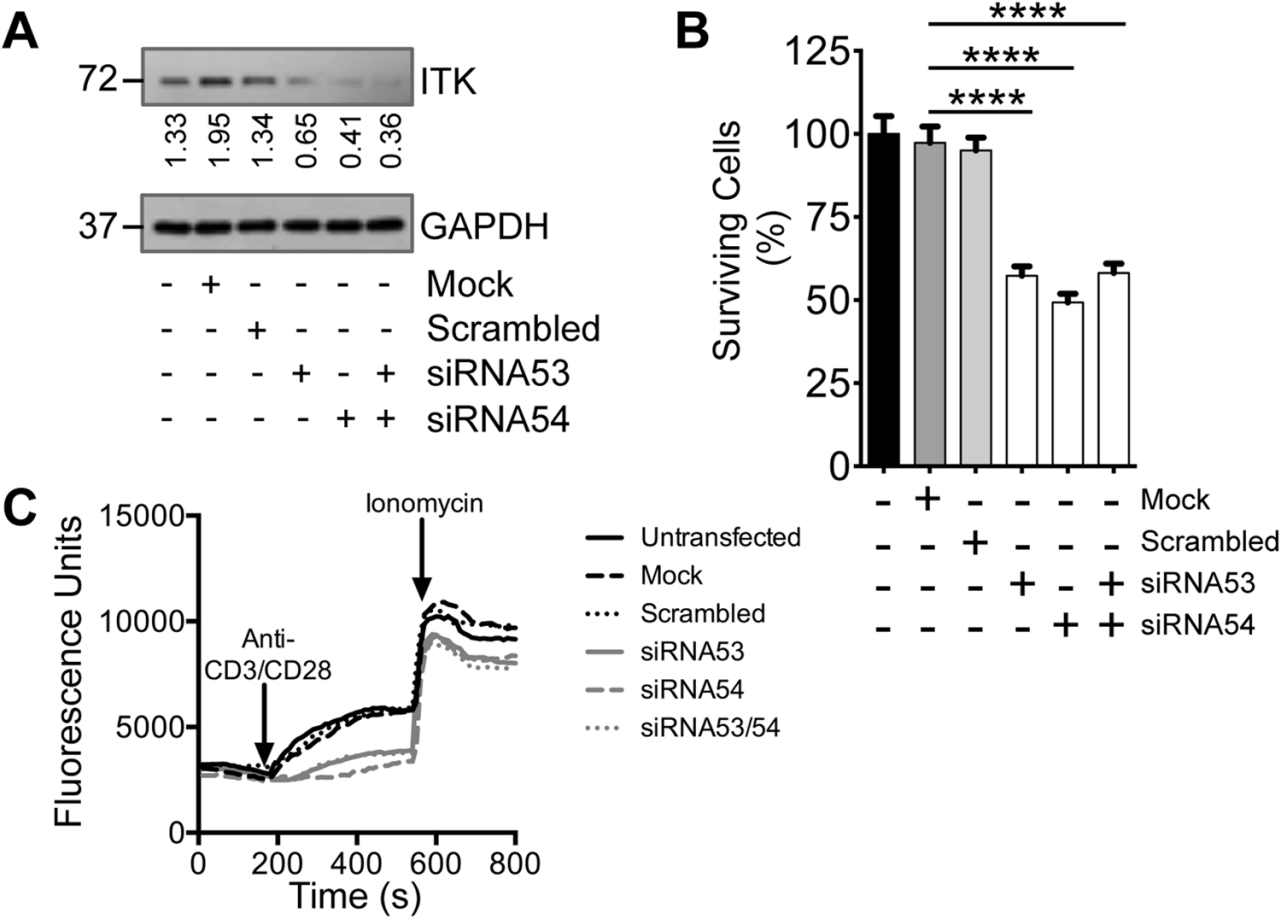
Effects of ITK knockdown on growth and calcium flux in Jurkat cells. (**A**) Westerns showing ITK knockdown due to two different siRNAs, 53 and 54, individually and in combination. Molecular weight is indicated to the left of the western. GAPDH is a loading control. (**B**) Cell survival measured as ATP luminescence following ITK knockdown by siRNAs, 53 and 54, individually and in combination. Results are mean ± SEM. n = 3. siRNA produce significant reductions in cell survival (two-tailed unpaired t-test). ****P < 0.0001. (**C**) Calcium flux changes in response to ITK knockdown. Black lines are fluorescence measurements following stimulation by anti-CD3 and anti-CD28 antibodies for untransfected cells (solid line), mock transfected (dashed line) or scrambled negative control siRNA (dotted line). Grey lines are fluorescence measurements for siRNA 53 (solid line), siRNA 54 (dashed line) or siRNAs 53 and 54 together (dotted line). Ionomycin was added at the indicated time.

As an independent means of assessing ITK function in Jurkat cells we carried out siRNA knockdown (Fig. 3A). This produced significant reduction in cell survival (two tailed unpaired t-test, *P* < 0.0001) (Fig. 3B) and effectively suppressed calcium flux (Fig. 3C).

### Comparative effects of ITKi on signalling and cytokine production

**Figure 4:**
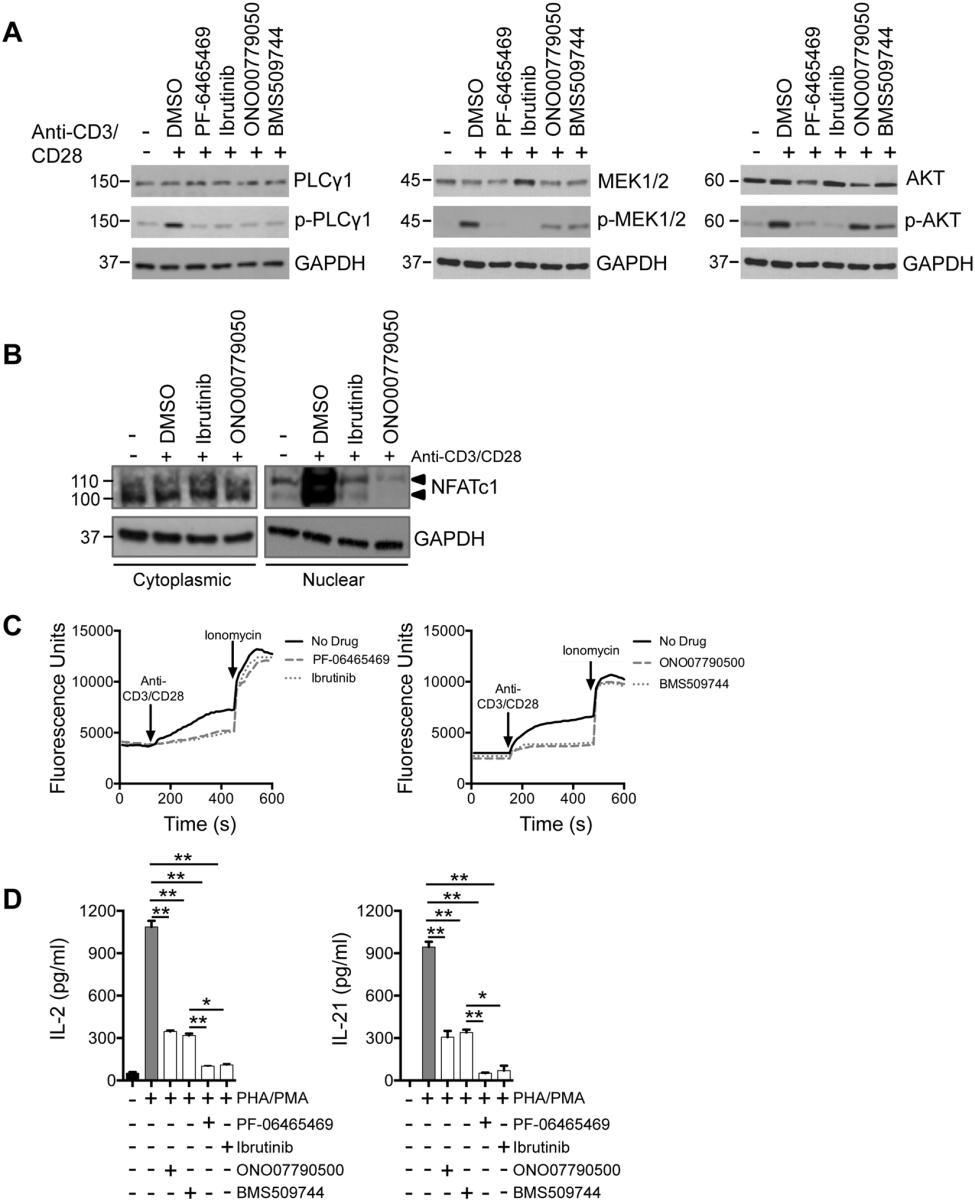
Effects of ITKi on signalling pathways, calcium flux and cytokine production in Jurkat cells. (**A**) Jurkat cells were stimulated with anti-CD3 and anti-CD28 antibodies as indicated. Westerns showing expression of PLCγ1 and phosphorylated-PLCγ1 (p-PLCγ1), MEK1/2 and phosphorylated-MEK1/2 (p-MEK1/2) and AKT and phosphorylated-AKT (p-AKT). GAPDH is a loading control. Molecular weight is indicated to the left of the western. (**B**) Jurkat cells were pre-treated with 1 µM ONO00779050 (1 µM) and Ibrutinib (1 µM) for 30 minutes, and then stimulated with anti-CD3/CD28 antibodies for 6 hours. Cytoplasmic and nuclear extracts were analysed by western blot. Arrowheads indicate NFATc1. GAPDH is a loading control. Molecular weight is indicated to the left of the western. (**C**) Calcium flux changes in response to ITKi. Left hand panel shows effect of PF-06465469 (dashed line) and ibrutinib (dotted line). Right hand panel shows effect of ONO7790500 (dashed line) and BMS509744 (dotted line). For each panel the no drug control is indicated by a solid line. The time of addition of anti-CD3 and anti-CD28 antibodies and ionomycin are shown. (**D**) IL-2 (left-hand panel) and IL-21 (right-hand panel) production in response to phytohaemagglutinin and phorbol myristate (PHA/PMA). Results are mean ± SEM. n = 4. IL-2 and IL-21 production were significantly (two-tailed unpaired t-test) repressed by ibrutinib, PF-6465469, BMS509744 and ONO7790500.

ITK is important in T-cell receptor and downstream signaling. All four ITKi abolished phosphorylation of PLCγ, which lies immediately distal to ITK in the signaling cascade (Fig. 4A) but there were differential effects on MEK1/2 and AKT. The more specific ITKi, ONO7790500 and BMS509744, did not prevent phosphorylation of MEK1/2 or AKT while PF-6465469 and ibutinib did have this effect. Effects on MEK1/2 and AKT signaling might be due to off-target effects of the less specific ITKi. ITK exerts many of its effects through nuclear translocation of the transcription factor, NFATc1. We demonstrated that stimulation of Jurkat cells by anti-CD3/CD28 caused nuclear translocation of NFATc1 and this was abolished by ITK inhibitors (Fig. 4B). Next we investigated effects of ITKi on calcium flux (Fig. 4C). All four inhibitors repressed calcium flux in response to anti-CD3/CD28 stimulation.

Cytokine production by T-cells is important for T-cell differentiation^37,38^ and germinal centre B-cell responses^14,39^. We demonstrated that Jurkat cells produce IL-2 and IL-21 following PHA/PMA stimulation. IL-2 production was significantly (two-tailed unpaired t-test) repressed by ibrutinib (*P* = 0.0034), PF-6465469 (*P* = 0.002), BMS509744 (*P* = 0.008) and ONO7790500 (*P* = 0.004) and the less specific inhibitors ibrutinib (*P* = 0.02) and PF-6465469 (*P* = 0.005) produced significantly more repression than ONO7790500 or BMS509744 (Fig. 4D). Similarly IL-21 production was significantly (two-tailed unpaired t-test) repressed by ibrutinib (*P* = 0.004), PF-6465469 (*P* = 0.003), BMS509744 (*P* = 0.004) and ONO7790500 (*P* = 0.006) and again the less specific inhibitors ibrutinib (*P* = 0.03) or PF-6465469 (*P* = 0.008) produced significantly more repression than ONO7790500 or BMS509744 (Fig. 4D).

### ITKi effects on viability and apoptosis

**Figure 5:**
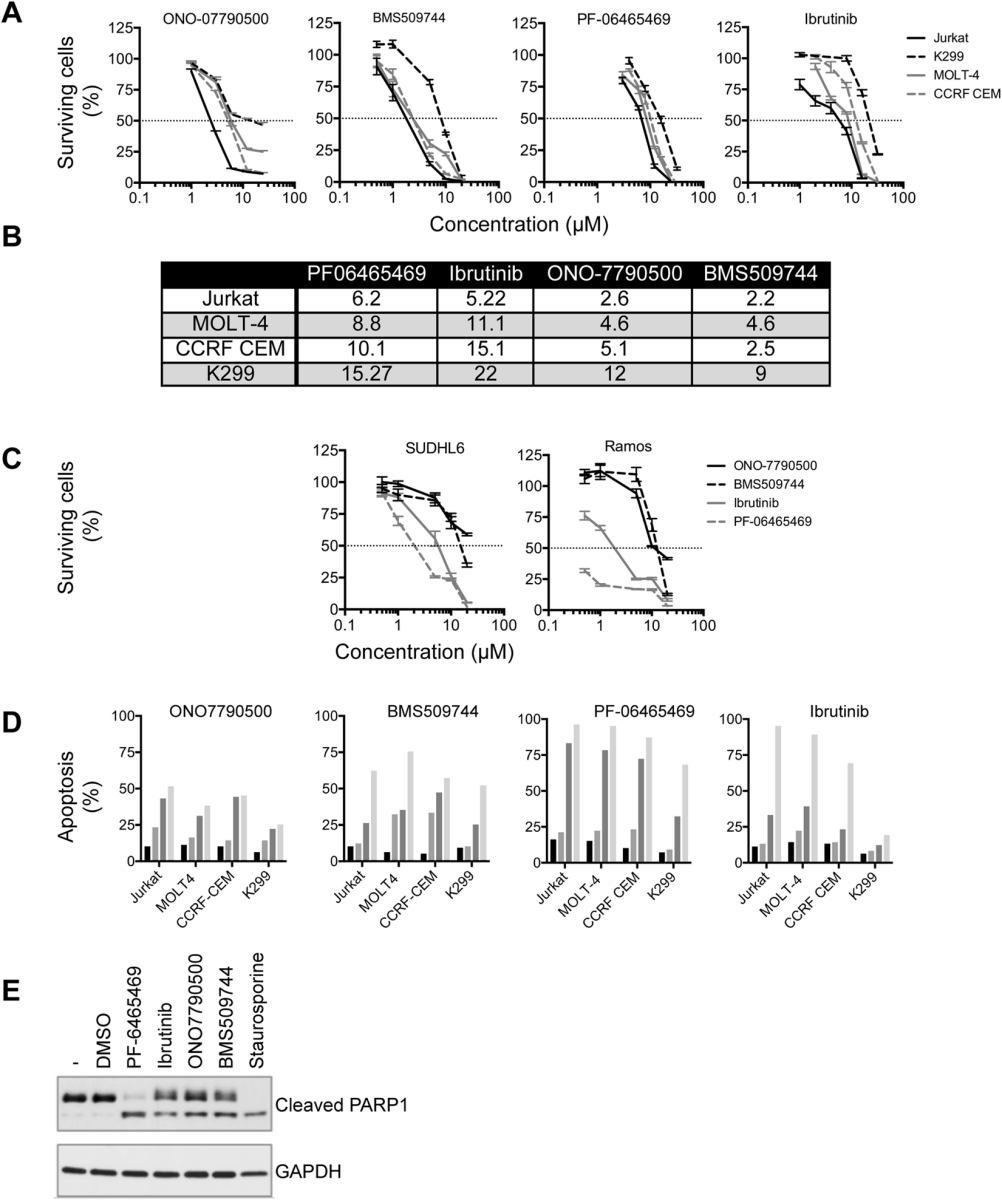
Effects of ITKi on survival and apoptosis of T-cell and B-cell lines. (**A**) Dose-survival curves for four T-cell lines (Jurkat, MOLT4, CCRF CEM and K299) as indicated in the legend to the right in response to ITKi as shown above each graph. The fraction (%) of cells surviving relative to T = 0 is shown. Mean ± SEM are shown. n = 3. 50% survival is indicated by the horizontal dotted line. (**B**) Table showing IC50 (µM) for each of the ITKi and each of the T-cell lines. (**C**) Dose survival curves for H460 (lung cancer cell line) and B-cell lymphoma cell lines, SUDHL6 and Ramos in response to ITKi as indicated in the legend to the right. Mean ± SEM are shown. n = 3. (**D**) Apoptosis induced by ITKi in the four T-cell lines. For each cell line apoptosis was measured at 4 concentrations of each ITKi (0, 4, 8 and 16 µM) after 24 hours. n = 2. (**E**) Westerns demonstrating PARP1 cleavage in Jurkat cells at T = 0 and after 24 hours with vehicle (DMSO) or ITKi (10 µM). Staurosporine (10 µM), a protein kinase inhibitor, was employed as a positive control. GAPDH is a loading control. Molecular weight is indicated to the left of the western. Representative of two independent experiments.

We determined the effects of ITKi on cell line viability as measured by ATP luminescence. For all four small molecules investigated Jurkat was the most sensitive cell line while K299 was the least sensitive (Fig. 5A). However, differences between IC50s for survival for these two cell lines (Fig. 5B) were modest at between 2.5-fold for PF-6465469 and 4.6-fold for ONO7790500. This could be due to effects on RLK, the TEC family kinase that is more highly expressed in K299 (Fig. 2C). Western blotting showed no detectable tyrosine phosphorylation of RLK at baseline and, therefore, there was no discernible effect of ITKi on tyrosine phosphorylation suggesting either that the small molecules have other effects on RLK or off-target effects (Supplemental Fig. 1). In order to assess further the off-target effects on survival of ITKi we investigated the non-small cell lung cancer cell line, H460, and two B-cell lymphoma cell lines, SUDHL6 and Ramos (Fig. 5C). All the inhibitors had IC50s >10 µM for H460 while for the B-cell lines, which express BTK, the ONO7790500 IC50 was >20 µM and BMS509744 IC50 was >10 µM while for PF-6465469 and ibrutinib IC50s were <10 µM. This suggests that ONO7790500 and BMS509744 had little activity against BTK whereas PF-6465469 and ibrutinib reduce survival through their effects on this enzyme.

ATP luminescence is not a measure of apoptosis and, therefore, we directly determined apoptosis in T-cell lines due to ITKi (Fig. 5D,E). Apoptosis at lower concentrations of ITKi (4 µM) varied from 10 to 30% across cell lines but was 40 to 93% at the highest concentration (16 µM). Apoptosis occurred in K299, a cell line without detectable ITK, to almost the same degree as the other cell lines. Analysis of PARP1 cleavage (Fig. 5E) confirmed that ITKi induced apoptosis with PF-6465469, one of the less selective inhibitors, appearing to be most effective.

**Figure 6:**
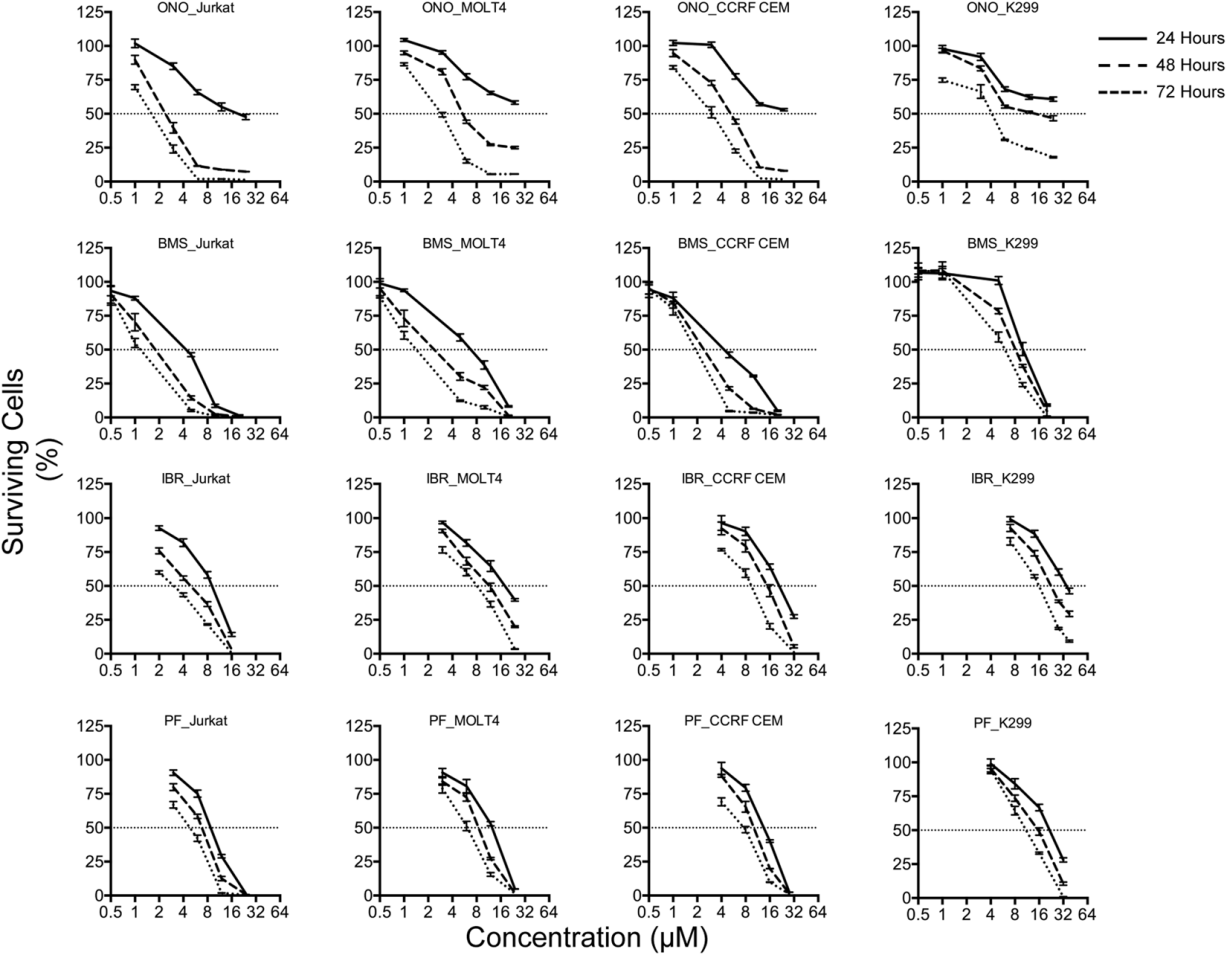
Dose and time dependence of survival responses of T-cell lines to ITKi. Percentage of cells surviving (mean ± SEM) is plotted against drug concentration (log2 scale). The inhibitor employed and cell line is indicated above each graph. Surviving cells are plotted at three time points, 24 hours (solid line), 48 hours (dashed line) and 72 hours (dotted line). 50% survival is indicated by the horizontal dotted line.

Concentration and time dependence (Fig. 6) of reduction in viability was investigated in order to determine the effects of ITKi in greater detail. The kinetics of loss of viability appear to be slower for ONO7790500 with only a modest loss of viability at 24 hours although the other inhibitors almost reached their maximum effects at this time point. Secondly, the maximum loss of viability obtained with ONO7790500 at each time point is less for the K299 cell line, which has least ITK, than for the other inhibitors.

Overall the ITKi employed in this study appear to cause greater reduction in survival and induction of apoptosis in the more highly ITK expressing cell lines but the differences are quite modest. In line with the findings of others in primary human T-cells^40^, ITKi were not highly effective at inducing apoptosis. ONO7790500 appears to show the greatest specificity for ITK expressing cells in reducing survival.

### Synergy with chemotherapy and PI3K inhibitors

**Figure 7:**
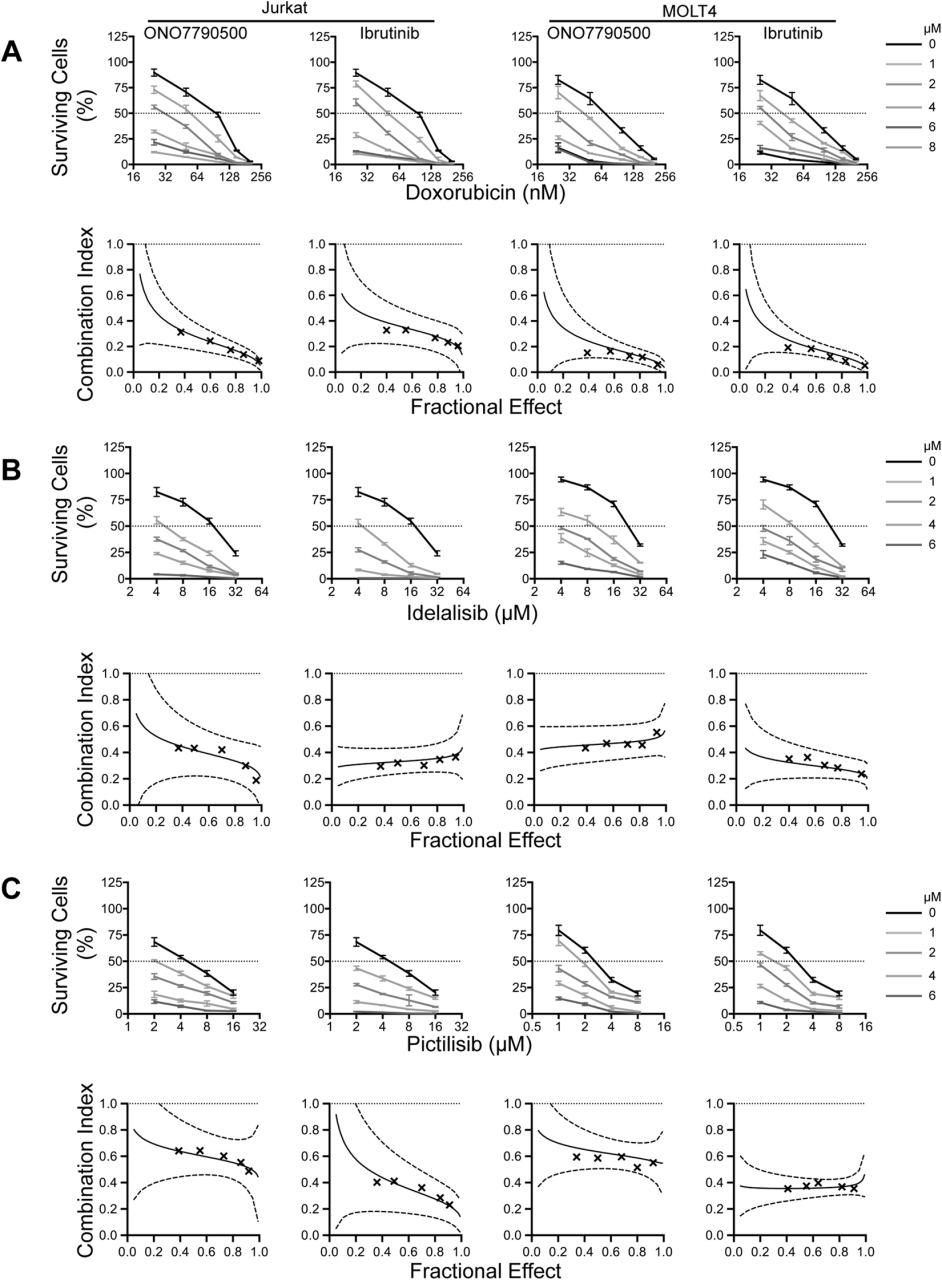
Synergy of ITKi with doxorubicin, idelalisib and pictilisib. Upper panels show survival (% of ATP luminescence at T = 0) of Jurkat cells (left-hand panels) or MOLT4 (right-hand panels) (mean ± SEM) cultured with varying combinations of drug and ITKi. Drug concentration is plotted on a log2 scale. Each line represents a different ITKi concentration, 0, 1, 2, 4, 6 and 8 µM, as indicated in the plot at top left of the figure. Lower panels demonstrate fractional effect (Fa)-combination index plots between the ITKi and doxorubicin in both Jurkat (left-hand panels) and MOLT4 (right-hand panels). The data points are plotted as crosses with the interpolation (solid line) and 95% confidence intervals (dashed lines). (**A**) Upper panels show survival of cells cultured with varying combinations of doxorubicin and ITKi. Lower panels demonstrate fractional effect (Fa)-combination index plots between the ITKi and doxorubicin. (**B**) Upper panels show survival of cells cultured with varying combinations of idelalisib and ITKi. Lower panels demonstrate fractional effect (Fa)-combination index plots between the ITKi and idelalisib. (**C**) Upper panels show survival of cells cultured with varying combinations of pictilisib and ITKi. Lower panels demonstrate fractional effect (Fa)-combination index plots between the ITKi and pictilisib.

Therefore, our studies of ITKi support the previously demonstrated key roles of this enzyme in calcium signaling and cytokine production^25,41^ but while we demonstrate that the most specific inhibitors, ONO7790500 and BMS509744, have very little activity against the BTK containing B-cell lines, Ramos and SUDHL6 (Fig. 5C), there is only modest distinction from K299, which expresses RLK but not ITK. Therefore, we investigated synergy with the anthracycline antibiotic, doxorubicin, and small molecule phosphoinositide-3-kinase (PI3K) inhibitors as routes to maintaining efficacy but avoiding off-target effects (Fig. 7). For these experiments we chose ONO7790500 and ibrutinib, which both have high affinity for ITK (Fig. 1) and two cell lines, Jurkat and MOLT4. Both ITKi synergised strongly with doxorubicin with combination indices (CI) <1 at all concentrations tested (Fig. 7A). Similarly both a specific PI3Kδ inhibitor, idelalisib, which is employed to treat chronic lymphocytic leukaemia^42^ and a non-specific inhibitor, pictilisib, which has been trialled in the treatment of breast cancer^43^ enhanced the effects of ITKi.

### ITKi impairs lymphocyte migration in combination with the CXCR4 antagonist, plerixafor

**Figure 8:**
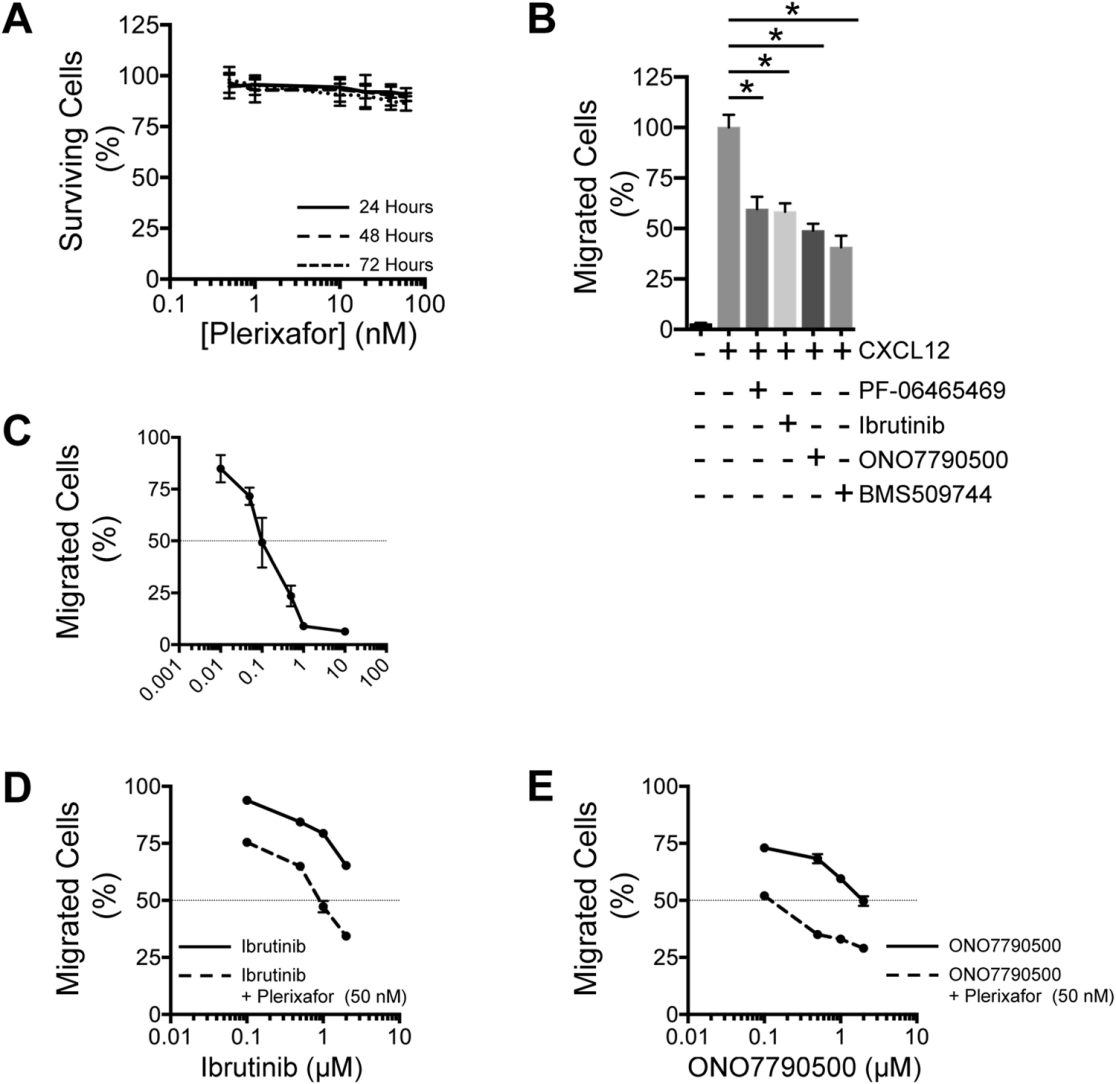
Synergistic inhibition of Jurkat cell migration. (**A**) Survival (% of ATP luminescence at T = 0) of Jurkat cells at various concentrations of plerixafor. Mean ± SEM. n = 3. Plerixafor concentration is plotted on a log10 scale. Survival at 24 hours (solid line), 48 hours (dashed line) and 72 hours (dotted line) are very similar. (**B**) Cell migration (% of cells placed in the top chamber at T = 0) in response to CXCL12 (100 ng/ml) and in the presence of vehicle only or ITKi as indicated. Mean ± SEM. n = 3. ITKi produced significant (unpaired 2-tailed t-test) reduction in migration. (**C**) Reduction in migration of Jurkat cells to CXCL12 by various concentrations of plerixafor. Mean ± SEM. n = 3. (**D**,**E**) Cell migration at various concentrations of ITKi (solid line) or ITKi with plerixafor (50 nM) (dashed line). ITKi concentration is plotted on a log10 scale. Mean ± SEM. The dotted horizontal line indicates 50% inhibition of migration. (**D**) Comparison of the effects of ibrutinib (solid line) with ibrutinib and plerixafor (dashed line). (**E**) Comparison of the effects of ONO7790500 (solid line) with ONO7790500 and plerixafor (dashed line).

Migration and invasiveness are key features of cancer^44^ and regulated trafficking of lymphocytes is essential for normal immunity. A small molecule inhibitor of lymphocyte egress from secondary lymphoid organs, FYT720, has potential uses in the treatment of multiple sclerosis^45^. We investigated the combination of ITKi with plerixafor, a molecule that is employed to harvest stem cells^46^. Firstly, we demonstrated that over a dose range (0.5 to 60 nM) plerixafor did not reduce cell viability at 24 or 48 hours demonstrating that cell death is not a cause for any differences in cell migration observed over this time (Fig. 8A). Next we demonstrated that ITKi significantly (unpaired 2-tailed t-test) inhibit migration of Jurkat cells in response to CXCL12; ONO7790500 (*P* = 0.01), BMS509744 (*P* = 0.01), ibrutinib (*P* = 0.02) and PF-06465469 (P = 0.03) (Fig. 8B). The more specific ONO7790500 and BMS509744 were slightly more effective although the difference between ibrutinib/PF-06465469 and ONO7790500/BMS509744 was not statistically significant. Plerixafor alone (Fig. 8C) reduced migration of Jurkat cells to CXCL12 with an EC50 of 100 nM. For synergy experiments we chose to employ plerixafor (50 nM), which alone reduced migration to 72%, in combination with a range of ITKi concentrations (Fig. 8D,E). Plerixafor enhanced the reduction in migration due to both ibrutinib and ONO7790500 (Fig. 8D,E). Plerixafor (50 nM) with either ibrutinib (1 µM) or ONO7790500 (0.1 µM) reduced migration to 50%. Overall the data suggests that ITK pathways are necessary for full migration of Jurkat cells in response to CXCL12 but are not sufficient.

## Discussion

In many cancers including PTCL there is a need for more effective therapies, which are also well-tolerated by patients. In addition resistance to chemotherapeutic agents is a major cause of treatment failure. The use of combination chemotherapy is a route to promoting efficacy while maintaining toxicities at tolerable levels and minimising opportunities for drug resistance. However, while there have been attempts to produce rational combinations with small molecule inhibitors^47^ there is at present no standard route to achieving this result.

There has been intense interest in Tfh signalling pathways since the discovery of the critical role of this subset in normal immunity^48^. Several surface molecules are targets for therapeutic antibodies. For example anti-PD-1 antibodies have been trialled in solid cancers^49^ and T-cell lymphomas^50^ and anti-ICOS antibodies are being investigated in the auto-immune condition, systemic lupus erythematosus^51^ as well as PTCL (clinicaltrials.gov identifier NCT02520791).

PI3K has a key role in Tfh cells^48^. Specifically the function of the isoform PI3Kδ has been investigated in T-cells in mice expressing catalytically inactive p110δ. T-cells in these animals demonstrated a failure of trafficking in response to T-cell receptor stimuli and subsequent failure of inflammation^52^. Further evidence for a specific PI3K role in T-cells comes from studies in which PI3Kδ was conditionally deleted from T- or B-cells^53^. PI3Kδ was required for formation of Tfh cells and specifically signalling from the surface protein, ICOS. Small molecule PI3Kδ inhibitors have entered clinical practice for the treatment of chronic lymphocytic leukaemia^42,54,55^. Overall inhibition of PI3K could be a strategy for T cell diseases.

The tumour microenvironment i.e. non-malignant stromal cells or lymphocytes and associated secreted growth factors and chemokines. supports proliferation and resistance to conventional chemotherapy in the B-cell lymphoproliferative disorders chronic lymphocytic leukaemia^56^ and follicular lymphoma^57^. The microenvironment is also likely to be important in peripheral T-cell lymphomas^58^. The G-protein coupled chemokine receptor, CXCR4, is highly expressed on normal germinal centre Tfh cells^59^. CXCR4 is associated with clinical outcome in acute myeloid leukaemia^60,61^ and melanoma^62^ demonstrating a wide biological role for CXCL12/CXCR4 signalling. Interruption of CXCR4 signaling by several methodologies has been suggested as a route to therapy^63–65^. More recently the farnesyl transferase inhibitor, tipifarnib, has been postulated to interrupt CXCL12/CXCR4 signalling in AITL^66^. Therefore, interruption of cell trafficking by disrupting CXCR4 signalling from the tumour microenvironment is a potential therapy in PTCL.

ITK is activated by CXCR4 in a PI3K dependent manner^22^ and it has been suggested that ITK inhibitors might be useful agents *in vivo* to perturb migration and could enhance the effects of cytotoxic chemotherapy. In this report we found that plerixafor, a CXCR4 antagonist already in clinical practice for mobilisation of stem cells prior to high dose therapy, enhanced the effects of ITK inhibitors in repressing migration of Jurkat T-cells while not having an effect on cell survival. An intriguing possibility is that adding Plerixafor and an ITK inhibitor to current regimens might enhance effectiveness by perturbing normal cell trafficking. Inhibition of lymphocyte migration is a target for therapy in the autoimmune condition multiple sclerosis^45^. Invasiveness is also a hallmark of cancer^44^. We postulate that disruption of lymphocyte trafficking will be an adjunct to current treatments for T-cell lymphoma.

We sought synergistic combinations with ITKi, which might have therapeutic usefulness. CHOP chemotherapy (of which the constituents are doxorubicin, cyclophosphamide, vincristine and prednisolone) is one of the standard treatment regimens for PTCL but therapeutic responses are not adequate or durable^4,67^. Our study demonstrated synergy between ITKi and doxorubicin. The PI3Kδi, idelalisib, is in clinical use for chronic lymphocytic leukemia^42^. We showed synergy between idelalisib or pictalisib, a non-specific PI3K inhibitor, and ITKi. Cell migration has not been a target for therapy but it is intriguing that plerixafor enhances the loss of migration caused by ONO7790500 or ibrutinib.

In summary, the combination of ITK inhibitors and some chemotherapeutic agents led to impressive effects *in vitro*. We suggest that ITK inhibitors might usefully be added to chemotherapy regimens for the treatment of some types of PTCL. Based on our preclinical results, validation of drug combination synergy between ITK inhibitors and conventional chemotherapies employed in this study needs to be assessed in animal models of lymphoma.

## Materials and Methods

### Cell lines and cell culture

Jurkat, MOLT-4, K299 and Ramos were maintained in RPMI-1640 (Invitrogen, Thermo Fisher Scientific, Waltham, MA, USA) with 10% fetal calf serum (FCS) (Lonza, Basel, Switzerland) and penicillin (10,000 units/ml) (Lonza) and streptomycin (10,000 g/ml) (Lonza) at 37 °C and 5% CO_2_. CCRF CEM cells were cultured in RPMI-1640 medium containing 20% FCS and penicillin (10,000 units/ml) (Lonza) and streptomycin (10,000 g/ml) (Lonza). HEK293 cells were maintained in DMEM medium (Invitrogen) and 10% FCS.

### siRNA knockdown of ITK

Jurkat cells were seeded in 24-well plates at a density of 4 × 10^5^ cells per well. On the day of transfection ITK specific siRNA s223953 and s223954 (Life Technologies, Thermo Fisher Scientific, Waltham, MA, USA) were employed singly or together in Opti-MEM reduced serum Medium (Invitrogen) at a final concentration of 100 nM with DharmaFECT1 transfection reagent (3 µl) (T-2001, Dharmacon, Lafayette, CO, USA). Efficiency of siRNA transfection in Jurkat cells was monitored using Block-iT™ Fluorescence Oligo (2013, Thermo Fisher Scientific).

### ITK Inhibitors

Four small molecule inhibitors of ITK were employed (Fig. 1). ONO-7790500 (IC50 <4 nM)(gift from Dr. Toshio Yoshizawa, ONO Pharmaceutical), BMS-509744 (IC50 19 nM) (5009, Tocris, Abingdon, UK), PF-06465469 (IC50 2 nM) (4710, Tocris) and ibrutinib (IC50 2.2 nM) (S2680, Selleckchem, Houston, TX, USA).

### Cell viability and apoptosis

Jurkat cell, K299, MOLT-4 and CCRF CEM lines were seeded in a flat-bottomed 96-well plate (Greiner, Kremsmünster, Austria) at 5 × 10^4^ cells/100 μl of medium per well and incubated with various concentrations of ITK inhibitors. Proliferation and viability were determined by CellTiter-Glo® (CTG) luminescent cell viability assay (Promega, Madison, WI, USA). Briefly, CTG reagent (100 μl) was added to 100 μl of cell suspension in a 96-well plate. After mixing on an orbital shaker to induce cell lysis for 2 minutes the plate was incubated at room temperature for a further 10 minutes. Luminescence readings were determined using the Wallac VICTOR^2^ Multilabel Counter (PerkinElmer, Beaconsfield, UK).

Apoptosis was determined using a FITC Annexin V Apoptosis Detection kit I (BD Biosciences, Wokingham, UK) and Draq7™ (BioLegend, San Diego, CA, USA). Cells were treated with ITK inhibitors at the indicated concentrations for 24 hours and pellets were harvested at 200 g for 5 minutes at room temperature, washed twice with cold PBS before resuspending in annexin binding buffer (100 µl) with annexin-V (5 µl) and Draq7 (1 µl). The cells were incubated for a further 20 minutes at room temperature before adding annexin binding buffer (400 µl) and incubating on ice. Data was acquired and analysed on a FACSCanto™ II ((BD Biosciences).

### Analysis of drug combinations

Jurkat cells were treated with five concentrations of the combination for 48 hours at 37 °C. The concentration ranges of drugs were based on the IC50 of these drugs. The anti-tumor activities of PF-06465469 or Ibrutinib in combination with Cyclosporine A (S2286, Sigma), Romidepsin (S3020, Sigma), Pralatrexate (S1497, Sigma), Gemcitabine (S1714, Sigma), Pictilisib (S1065, Sigma) and Doxorubicin (44583, Sigma) were assayed in 96-well plates.

The fractional affected (Fa) was calculated from the percentage growth inhibition by the formula Fa = 1 − (% growth/100). The Fa values were entered separately into Calcusyn (Biosoft, Cambridge, UK) analysis program for single agents and combinations. This method provides quantification of synergy (combination index, CI <1) or antagonism (CI >1) at different dose and effect levels.

### Calcium flux assay

Intracellular calcium mobilisation was detected with FluoForte Calcium Assay Kit (ENZ-51016, Enzo Life Sciences, Farmingdale, NY, USA). Jurkat cells (1.25 × 10^5^ cells/ml) were treated with ITK inhibitors at concentrations around the IC50 for growth for 4 hours at 37 °C. Cells were centrifuged at 800 g for 2 minutes and the pellets re-suspended in FluoForte dye-loading solution, and plated in 96-well black tissue culture plates (655086, Greiner). Cell plates were incubated for 45 minutes at 37 °C and then for 15 minutes at room temperature before activating by adding Dynabeads™ Human T-Activator anti-CD3/CD28 beads (Gibco, Thermo Fisher Scientific, Waltham, MA, USA). At the end of the experiment ionomycin (1 µg) (I3909, Sigma) was added to ensure maximal calcium mobilisation. Fluorescence was measured at excitation = 490 nm/emission = 525 nm with an Infinite M200 PRO (Tecan, Männedorf, Switzerland).

### RT-PCR

Total mRNA was extracted from harvested cells using a RNeasy Mini Kit (74104, Qiagen, Hilden, Germany). Reverse transcription (RT) was carried out with the SensiFAST^TM^ cDNA synthesis kit using the manufacturer’s protocol (Bioline, London, UK). A 20 µl RT reaction included 50 ng of total RNA, 5x TransAmp buffer (4 µl), reverse transcriptase (1 µl) and DNase/RNase free-water up to 20 µl. Taqman primers for ITK (Hs00950634_m1), RLK (Hs00177433_m1) and HPRT (Hs02800695) were purchased from Applied Biosystems (Foster City, CA, USA).

### Western blot analysis

Jurkat, K299, CCRF CEM and MOLT-4 cell lines were lysed with radioimmunoprecipitation-RIPA buffer (Tris-HCl pH 8.0 (50 mM), NaCl (150 mM) 1% sodium deoxycholate, and 0.1% SDS) supplemented with protease and phosphatase inhibitors (Sigma). Total cell lysates were incubated on ice for 10 to 15 minutes and centrifuged at 15,000 × g for 10 minutes at 4 °C to remove debris. Bicinchoninic acid (BCA) assay (Sigma) was used to quantify protein concentration.

For cytoplasmic lysates, Jurkat cells were resuspended in lysis buffer (Hepes pH 7.6 (20 mM), 20% glycerol, NaCl (10 mM), MgCl_2_ (1.5 mM), EDTA (0.2 mM), 0.1% TritonX-100, acetone (1 mM), DTT (1 mM) and orthovanadate (2 mM) along with the protease inhibitor cocktail (ab201111, Abcam)) and incubated with rotation at 4 °C for 15 minutes. The nuclei were pelleted (400 g at 4 °C for 10 minutes), resuspended in nuclear extraction buffer (Hepes pH 7.6 (20 mM), 20% glycerol, NaCl (250 mM), MgCl_2_ (1.5 mM), EDTA (0.2 mM), 0.1% TritonX-100, acetone (1 mM), DTT (1 mM) and orthovanadate (2 mM) with the protease inhibitor cocktail (ab201111, abcam)), and incubated with rotation at 4 °C for 1 hour. Nuclear extracts were collected by centrifugation at 16000 g at 4 °C for 10 minutes.

Lysates were separated by SDS-PAGE on 7.5% Mini-protean TGX Precast gels (BioRad, Hercules, CA, USA), and transferred to a polyvinyldine difluoride membrane (Mini Format, 0.2 µM PVDF Single Application, BioRad). The blot was incubated in TBST (Tris-HCl pH 7.6 (20 mM), NaCl (136 mM), and 0.1% Tween-20) supplemented with 5% skimmed milk (Oxoid, Thermo Fisher Scientific, Waltham, MA, USA) for 1 hour at room temperature. The membrane was incubated with primary antibody in blocking solution at 4 °C overnight. Primary antibodies used (all at 1:1000 unless otherwise stated) were anti-ITK (ab32039, Abcam, Cambridge, UK), anti-phospho-BTK/ITK (14-9015-82, eBioscience, San Diego, CA, USA), anti-PLCγ1 (#5690, Cell Signaling Technology, Danvers, MA, USA), anti-phospho-PLCγ1 (#2821, Cell Signaling Technology), anti-AKT (#4691, Cell Signaling Technology), anti-phospho-AKT (#4060, Cell Signaling Technology), anti-MEK (#8727, Cell Signaling Technology). anti-phospho-MEK (#9154, Cell Signaling Technology) and anti-NFAT2 (NFATc) (1 µg/ml) (ab2796, Abcam). Loading control was anti-GAPDH (1:10000) (#2118, Cell Signaling Technology). Secondary antibodies used were anti-mouse-IgG-HRP conjugated (1:2000) (#7076, Cell Signaling Technology) or anti-rabbit-IgG-HRP conjugated (1:2000) (#7074, Cell Signaling Technology).

After 3 to 5 washes with TBST, the blot was incubated with secondary antibody with shaking for 1 hour at room temperature. Blots were washed five times for 10 minutes, and signal was detected with chemiluminescent HRP substrate (BioRad) and imaged (SRX-101A X-Ray Film Processor, Konica Minolta, Bloxham Mill, UK) using medical X-ray film (Fujifilm, Tokyo, Japan).

To investigate RLK in K299 we used anti-RLK antibody (ab37818, Abcam) and anti-phospho-tyrosine antibody (4g10) (#9411, Cell Signaling Technology). Following incubation with both anti-mouse IgG (926–32212, LICOR Biosciences) and anti-rabbit IgG (#5366, Cell Signaling Technology) secondary antibodies at 1:10000 for 1 hour at room temperature fluorescence was detected using a LICOR-Odyssey machine (LICOR Biosciences, Lincoln, NE, USA).

### Chemotaxis Assay

A 24-well transwell chamber with polycarbonate membrane (5 µm) (catalog no. 3421; Costar, Kennebunk, ME, USA) was pre-coated with fibronectin (10 µg/ml) (F1141-1MG, Sigma-Aldrich). Jurkat cells were pretreated with ITK inhibitors (2 µM) for 24 hours in serum-free RPMI 1640 media. Chemotaxis medium (RPMI 1640 medium containing 25 mM HEPES buffer and 1% BSA) with and without CXCL12 (100 ng/ml) (350-NS-010, R&D Systems, Minneapolis, MN, USA) was added to the lower chamber with ITKi. Jurkat cell suspension (10^6^ cells in 100 µl) were placed in the upper wells. The cells were allowed to migrate for 3 hours at 37 °C. Migrated cells were collected in the lower compartment stained with trypan blue (BioRad) and counted by hemocytometer or automated cell counter.

### Statistics

Statistical analyses were undertaken using the GraphPad Prism version 7.00 (GraphPad Software, San Diego, CA, USA). Differences were considered to be significant when *P* < 0·05.

## Electronic supplementary material

Supplementary Information
