# Supplementary material for: Comparison of interleukin-2-inducible kinase (ITK) inhibitors and potential for combination therapies for T-cell lymphoma
Source: Sci Rep. 2018 Sep 21;8:14216. doi: 10.1038/s41598-018-32634-5 (PMC6154993; doi:10.1038/s41598-018-32634-5)
Supplement: Supplementary file 1 — Supplementary Information [file 41598_2018_32634_MOESM1_ESM.pdf]

Supplementary Information

Comparison of interleukin-2-inducible kinase (ITK) inhibitors and potential for combination therapies for T-cell lymphoma.

Sami Mamand, Rebecca L. Allchin, Matthew J. Ahearne and Simon D. Wagner

Supplemental Figure 1

RLK protein expression in K299. Western blots probed with anti-RLK (left-hand panel) and simultaneously with anti-phospho-tyrosine and anti-GAPDH (right-hand panels). Molecular weight is indicated to the left of each blot. The expected molecular weight of RLK is ~61 kDa as indicated by the blue arrowheads.

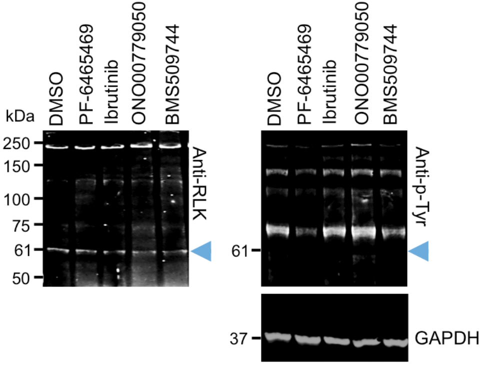

Mamand et al.
Supp. Fig 1

Transparency Information

Figure 2A

Jurkat and K299 cell lines

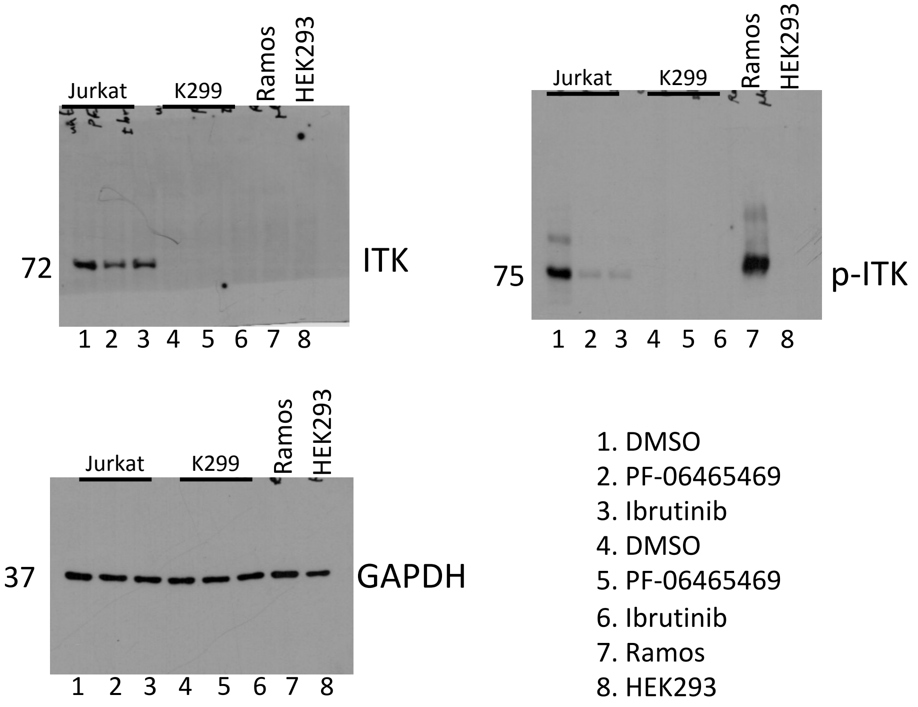

1. DMSO
2. PF-06465469
3. Ibrutinib
4. DMSO
5. PF-06465469
6. Ibrutinib
7. Ramos
8. HEK293

Figure 2A

MOLT-4 and CCRF cell lines

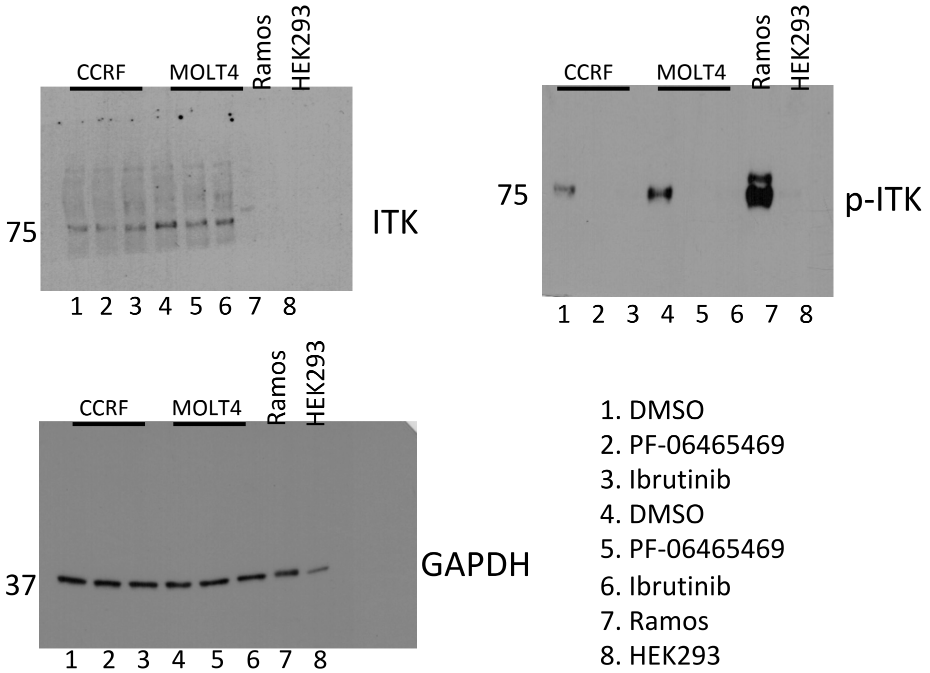

1. DMSO
2. PF-06465469
3. Ibrutinib
4. DMSO
5. PF-06465469
6. Ibrutinib
7. Ramos
8. HEK293

Figure 2B

Jurkat and K299 cell lines

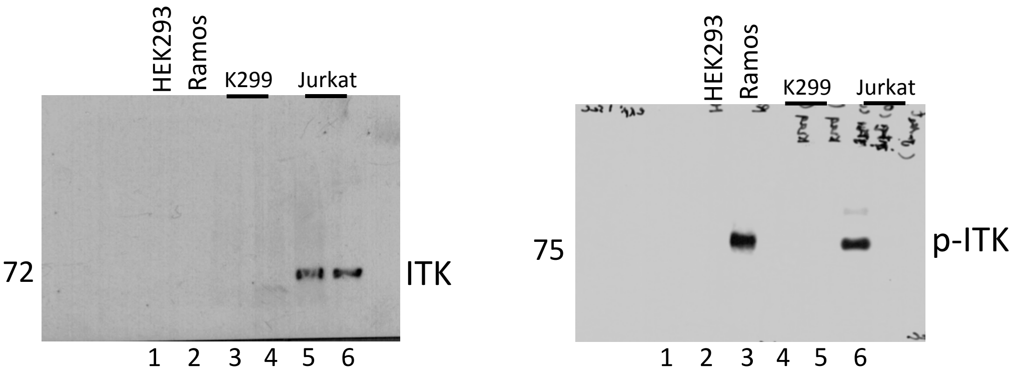

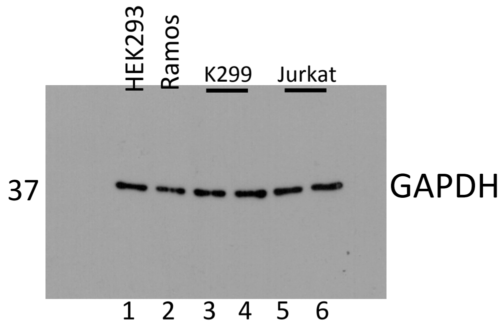

1. HEK293
2. Ramos
3. DMSO
4. ONO7790500
5. DMSO
6. ONO7790500

Figure 2B

MOLT-4 and CCRF cell lines

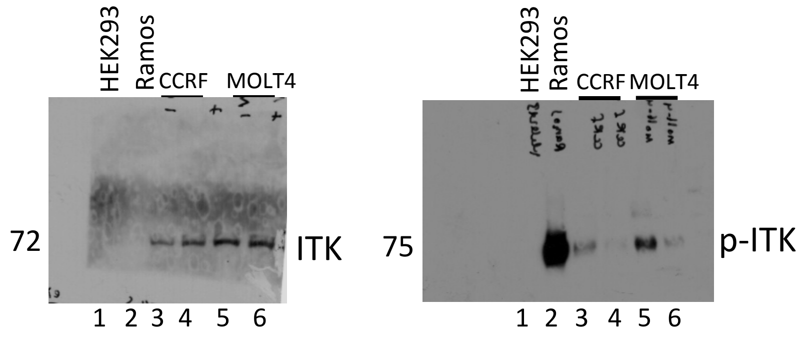

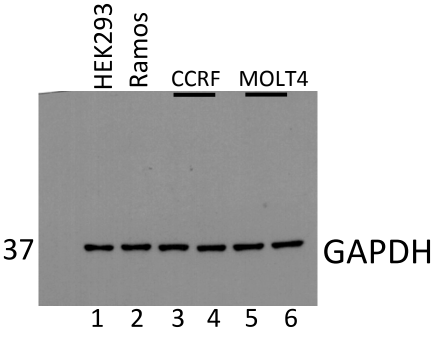

1. HEK293
2. Ramos
3. DMSO
4. ONO7790500
5. DMSO
6. ONO7790500

Figure 2B

Jurkat and K299 cell lines

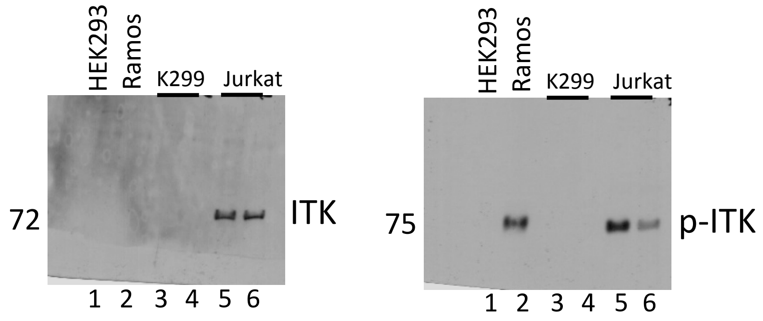

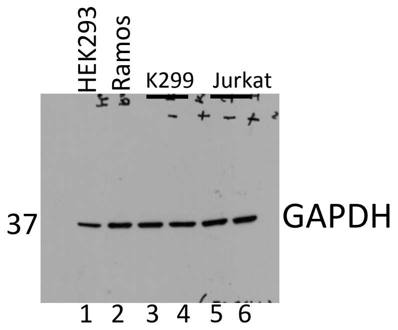

1. HEK293
2. Ramos
3. DMSO
4. BMS509744
5. DMSO
6. BMS509744

Figure 2B

MOLT-4 and CCRF cell lines

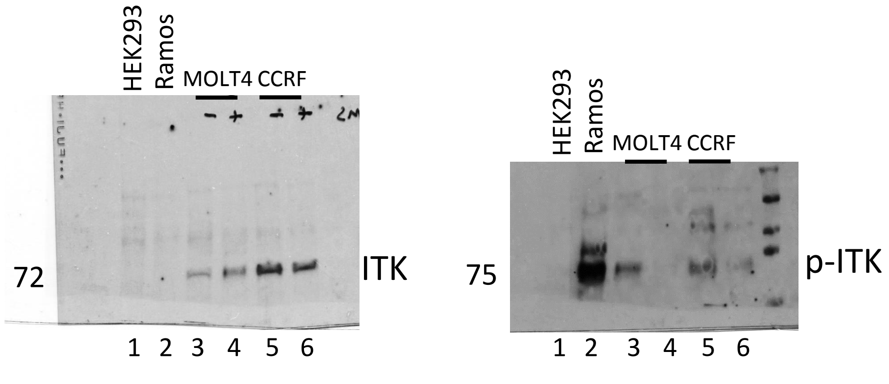

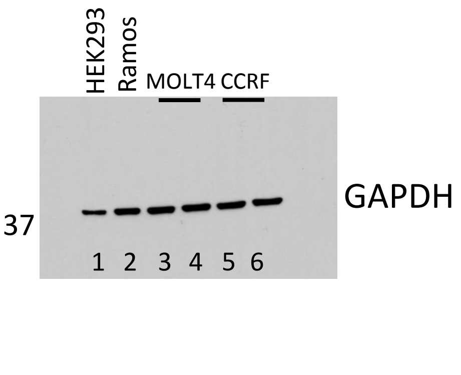

1. HEK293
2. Ramos
3. DMSO
4. BMS509744
5. DMSO
6. BMS509744

Figure 3A

ITK-siRNA Knockdown

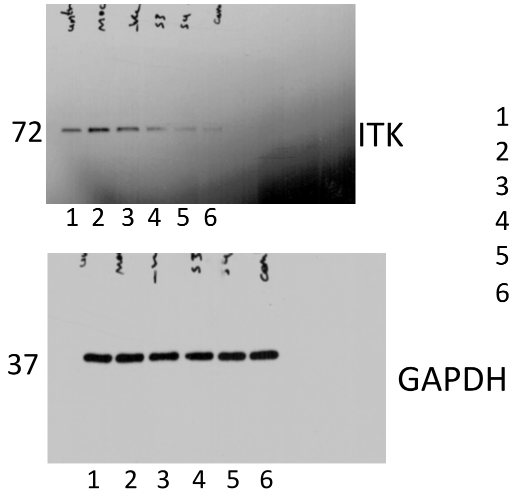

1. Untrans
2. Mock
3. Scrambled
4. S223953
5. S223954
6. S223953/54

Figure 5E

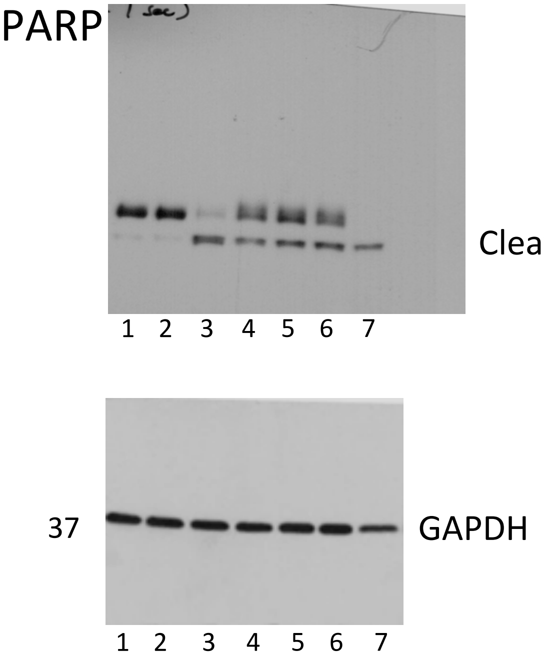

1. Untreated
2. DMSO
3. PF-6465469
4. Ibrutinib
5. ONO00779050
6. BMS509744
7. Staurosporine

Figure 4A

AKT, MEK1/2 and PLCγ1

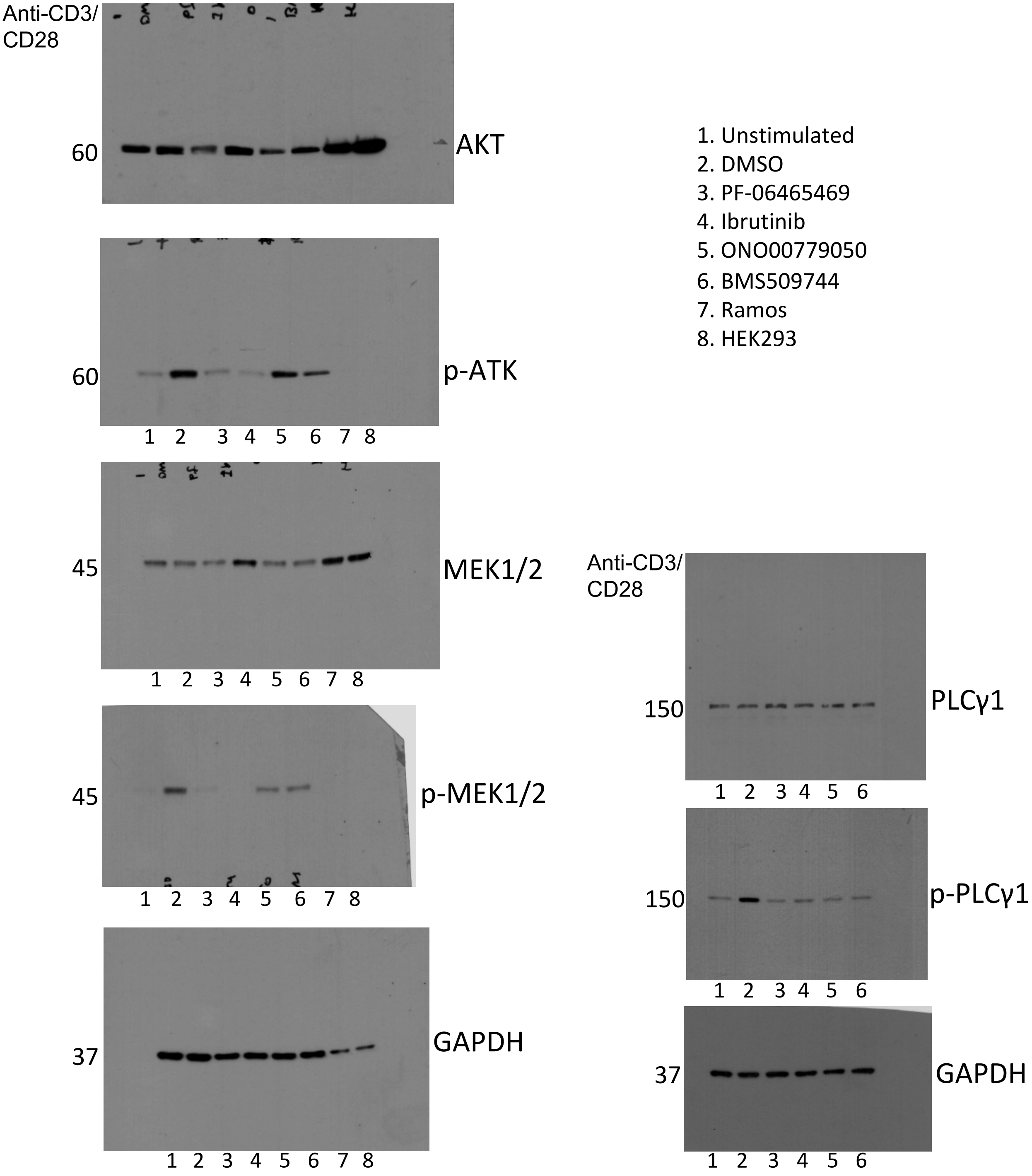

Figure 4B

Cytoplasmic-NFATc1

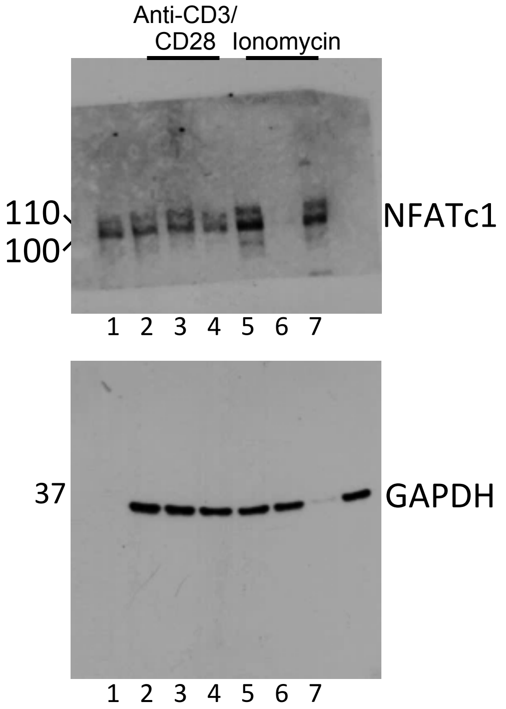

1. Untreated
2. DMSO
3. Ibrutinib
4. ONO00779050
5. DMSO
6. Ibrutinib
7. ONO00779050

Figure 4B

Nuclear-NFAT

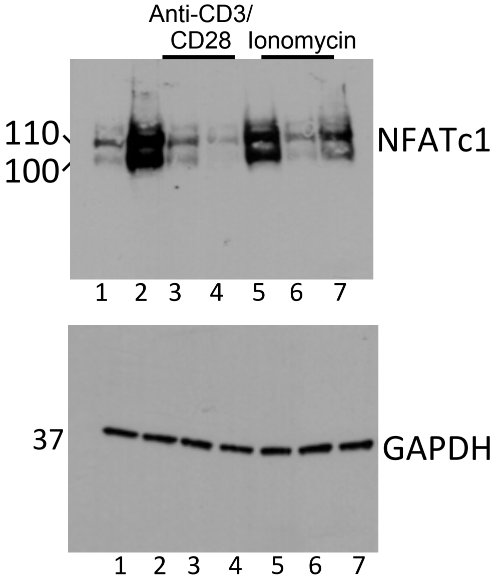

1. Untreated
2. DMSO
3. Ibrutinib
4. ONO00779050
5. DMSO
6. Ibrutinib
7. ONO00779050
